# Does bunion surgery actually narrow the foot? Assessment of outcomes of surgery using traditional angles and a new radiographic measure of severity- the forefoot: hindfoot ratio. Correlation with clinical outcomes

**DOI:** 10.1186/1757-1146-8-S1-A15

**Published:** 2015-04-20

**Authors:** Sarah Curran, Ashok Marudanayagam, Laura Beddard, Anthony Perera

**Affiliations:** 1 Department of Healthcare, Cardiff Metropolitan University, Cardiff, UK; 2 Cardiff and Vale University Health Board University Health Board, Cardiff, UK; 3 Spire Cardiff Hospital; Cardiff, UK; 4 London Foot and Ankle Centre, London, UK

## Background

Various angles have been used to grade the severity of hallux valgus deformity. They are useful in surgical planning but do not correlate with symptom severity or improvement. We feel that there is a fundamental mismatch between the width of the forefoot and the width of the hindfoot and that this is more clinically relevant, we describe two techniques for measuring this. We aim to measure the degree of foot narrowing after surgery and moreover how this correlates to the severity of pre- and post operative outcomes.

## Methods

200 consecutive bunion operations were assessed with weight bearing radiographs. The HVA and IMA were measured according to standard practice. We also assessed forefoot width using two methods we have described. The first is the ‘Forefoot Width’ measured as a perpendicular to the midfoot (a technique we have previously validated). The ‘Foot Ratio’ is calculated as a function of the calcaneal width. Clinical outcomes were assessed using the Manchester Oxford Foot Questionnaire (MOXFQ), American Orthopaedic Foot and Ankle Surgeons (AOFAS) questionniare.

## Results

Bunion surgery narrows the osseous width of the forefoot. This narrowing can be by as much as 23mm in cases with severe deformity. We found that the Forefoot: Hindfoot ratio correlated with symptom severity and that normalisation of the ratio to below 2.5 was associated with better outcomes. This is important as small absolute corrections were associated with good outcomes.

## Conclusion

Our measure of Forefoot Width is reproducible and allows for variations such as forefoot adductus. We feel that the Forefoot:Hindfoot ratio is more important as this determines the ability to fit into off-the-shelf footwear rather than requiring bespoke or modified footwear. This is the first study to look at the ability to narrow the forefoot and has important implications in determining patient selection and post-operative outcomes.

